# Antioxidant Potential of *Vespa affinis* L., a Traditional Edible Insect Species of North East India

**DOI:** 10.1371/journal.pone.0156107

**Published:** 2016-05-19

**Authors:** Prachurjya Dutta, Tapan Dey, Prasenjit Manna, Jatin Kalita

**Affiliations:** 1 Academy of Scientific and Innovative Research, Chennai– 600113, India; 2 Biological Science and Technology Division, CSIR-North East Institute of Science and Technology, Jorhat, Assam, 785006, India; 3 Dibrugarh University, Dibrugarh, 786004, Assam, India; Jadavpur University, INDIA

## Abstract

**Introduction:**

Elevated oxidative stress plays an important role in the pathogenesis of health disorders, like arthritis. Traditionally, *Vespa affinis* L., a common edible insect among many tribes in North-East India, is believed to have a beneficial role in extenuating health disorders, such as arthritis. The present study investigated the molecular mechanism underlying medicinal benefit of the Aqueous Extract of *Vespa affinis* L. (AEVA) against oxidative stress pathophysiology.

**Methods:**

The free radical scavenging activities of AEVA were examined against DPPH, hydroxyl, and superoxide radicals and the effect on the activities of antioxidant enzyme (GST and CAT) was determined using both recombinant proteins and human plasma. The antioxidant potential of AEVA was again investigated using THP-1 monocytes.

**Results:**

AEVA possesses a significant free radical scavenging activity as evident from the DPPH, superoxide, and hydroxyl radical scavenging assay. Incubation of AEVA (2.5, 5, 7.5, and 10 μg/μL) with the recombinant antioxidant enzymes, rGST and rCAT significantly increased the enzyme activities compared to those observed in corresponding enzyme alone or AEVA itself. AEVA supplementation (5, 7.5, and 10 μg/μL) also stimulates the activities of GST and CAT when incubated with human plasma. A cell culture study also confirmed the beneficial role of AEVA (0.8 and 1.2 μg/μL) which enhances the activities of GST and CAT, and also reduces the intercellular ROS production in monocytes treated with or without H_2_O_2_ and the effects are at par with what is observed in N-acetyl cysteine-treated cells.

**Conclusion:**

The antioxidant potential of the aqueous extract of *Vespa affinis* L. may mediate its therapeutic activities in oxidative stress-associated health disorders.

## Introduction

The practice of eating insects as food is well known among different parts of the world [1]. From long decades people around the world have been eating insects as a regular part of their diets. It has been proven that edible insect species contain significant amount of protein, essential amino acids, and other micro nutrients [2–4]. Apart from its nutritional aspects, many insect species have been found to use for the treatment of different diseases, such as wound healing, respiratory disorder, paralysis, whooping cough, anemia, etc. [5]. The term, entomotherapy is known as the uses of insects for the treatment of several diseases [6]. Bednarova et al. [7] reported that eating of few edible insects was beneficial for people suffering with hyperuricemia or gout because these food items are the sources of low-purine and protein-rich diet.

The prevalence of oxidative stress has been implicated in the pathogenesis of various diseases, such as arthritis, diabetes, cardiovascular complications, cancer, etc. [8, 9]. Both clinical and experimental studies in the literature suggested the benefits of antioxidant supplements as complementary therapy for the treatment of oxidative stress-associated health disorders [10]. Beneficial role of herbal antioxidants has been well documented in preventing the free radical-mediated organ pathophysiology [11]. However there is no report investigating the antioxidant property of the edible insect species. The larvae and pupae of *Vespa sp*. (Hymenoptera: Vespidae) is consumed by various tribes and communities in North-East India [6, 12, 13]. The nutritional value of this species has been reported as 50.13 g of protein, 13.29 g of carbohydrate, and 25.33 g of fats per 100 g species [14]. Senthilkumar et al. [5] reported that this insect species has been used traditionally for the treatment of arthritis. So far no mechanistic study has been carried out to investigate the beneficial property of this insect species against oxidative stress.

The aim of the present study is to investigate the antioxidant potential of the aqueous extract of *Vespa affinis* L. (AEVA). Using human plasma, recombinant enzymes, and human THP-1 monocytes, results showed that AEVA possesses significant radical scavenging potential and stimulates the activities of antioxidant enzymes (GST and CAT). Results suggest that AEVA could be used as a beneficial therapeutic against oxidative stress-associated health disorders.

## Materials and Methods

The study was conducted at CSIR-North East Institute of Science and Technology, Jorhat, Assam after ethical clearance from the Institutional Ethics Committee, NEIST, Jorhat.

### Chemicals

Bradford reagent, 2,2-Diphenyl-1-picrylhydrazyl **(**DPPH), 1-chloro-2,4-dinitrobenzene (CDNB), ethylene diaminetetraacetic acid (EDTA), glacial acetic acid, hydrogen peroxide(H_2_O_2_), ferric chloride (FeCl_3_), nicotinamide adenine dinucleotide reduced (NADH), nitroblue tetrazolium (NBT), phenazine methosulphate (PMT), RPMI-1640 medium, recombinant glutathione *S*-transferase (rGST), recombinant catalase (rCAT), sodium pyrophosphate, reduced glutathione (GSH), thiobarbituric acid (TBA), ascorbic acid, trolox, potassium dihydrogen phosphate (KH_2_PO4), dithiothreitol (DTT), ethylenediaminetetraacetic acid (EDTA), sodium sulfate (Na_2_SO_4_), anthrone reagent, sulphuric acid (H_2_SO_4_), vanillin reagent, ortho-phosphoric acid (H_3_PO_4_), chloroform, methanol, triolein, D-Glucose, and DCFDA (2´, 7´-Dichlorofluoresceindiacetate) were purchased from Sigma-Aldrich Chemical Company (St. Louis, MO, USA).

### Sample Collection

*Vespa affinis* L. belonging to the family of Vespidae (Hymenoptera) a common edible insect species of North-East India has been taken for the present investigation. The pupae were collected during the month of August-September from the Surusarai Tinali market of Jorhat District, Assam, India (Geographic coordinates: ~26^0^44′45.4″ North and 94^0^08′47.6″ East). The bodies were cleaned with distilled water and kept at -45°C until further experiments.

### Proximate composition of nutritional value in *V*. *affinis* L.

The protein content was determined by Bradford Assay using BSA as standard [15]. The lipids content was determined by phospho-vanillin Assay using triolein as standard [16]. All carbohydrates including glycogen and the soluble carbohydrates were determined by classical colorimetric method based on anthrone reagent using D-Glucose as standard [17]. The ash content was determined by heating in a muffle furnace (Ikon Instruments) at 600°C to constant weight [18]. The moisture contain was determined according to the method of Hart and Fisher [19]. Total thiol content was estimated using the kit from Cayman Chemical (MI, USA).

### Preparation of aqueous extract of *V*. *affinis* L. (AEVA)

Aqueous extract was prepared following the method as described elsewhere [20]. About 10g pupae of *V*. *affinis* L. were homogenized with 40 mL of distilled water at 4°C. The mixture was then centrifuged at 7000 rpm for 30 minutes to get rid of unwanted debris. The supernatant was collected, stored at -80°C, and used for this study.

### Chemical profiling of AEVA

The infrared (IR) and mass spectra (MS) analyses were performed to examine the chemical profiling of AEVA [21]. The IR spectrum analysis was done by the KBr pellet technique using FTIR instrument (Shimadzu). The MS analysis was performed on an ESI-Q-TOF micro mass spectrometer (Waters) in the electrospray ionization mode (positive mode).

### Blood sample collection

Blood samples were collected from fasting healthy adult volunteers in the EDTA tubes at Clinical Centre, CSIR-NEIST, Jorhat, Assam after receiving written consent according to the protocol approved by Indian Council of Medical Research (ICMR). The Institutional Ethical Committee (IEC) of North East Institute of Science and Technology, Jorhat, Assam has approved (NEIST/ICE/2008/2009, dated 3^rd^ March 2009) the protocol for blood collection from volunteers. Clear plasma was separated by centrifugation of the blood at 3000 rpm for 15 min.

### Determination of the radical scavenging activity of AEVA in cell-free system

#### Quenching of DPPH radical

The DPPH radical scavenging activity of AEVA was measured following the method reported earlier [22]. Various concentrations of AEVA (0.25, 0.5, 0.75, 1.25, 2.5, 3.75, 5, and 6.25 μg/μL) were added to DPPH solution in methanol (125 μM, 1 mL). The solution was shaken and incubated at 37°C for 30 minutes in dark. The final volume was adjusted to 2 ml by adding water. The decrease in absorbance was measured at 517 nm against methanol blank using microplate reader (BioTek, USA). Percent inhibition was calculated by using the equation, I = (A_0_-A_1_/A_0_) × 100, where A_0_ is the absorbance of the blank and A_1_ is the absorbance of test sample. Ascorbic acid was used as a positive control.

#### Quenching of hydroxyl radical

The hydroxyl radical scavenging activity of AEVA was examined by using the 2-deoxyribose oxidation method [23]. 2-Deoxyribose is oxidized by the hydroxyl radical generated by Fe^3+^/Ascorbate/EDTA/H_2_O_2_ system (Fenton reaction) and degraded to malondialdehyde. The extent of deoxyribose degradation was measured by TBA method. The reaction mixture contained 2-deoxy-D-ribose (2.8 mM), FeCl_3_ (100 μM), EDTA (104 μM), and various concentrations (1.25, 2.5, 5, 7.5, 10, 12.5, and 15 μg/μL) of AEVA. The final volume was adjusted upto1 mL by adding phosphate buffer (20 mM, pH 7.4). The reaction was started by the addition of H_2_O_2_ (1 mM). After incubation for 1 hr at 37°C, 1 ml of TBA (1%) was added to the reaction mixture and further incubated at 100°C for 20 min. Finally, the solution was ice cooled, centrifuged at 5000 rpm for 15 min, and absorbance of the supernatant was measured at 530 nm using microplate reader (BioTek, USA). The control was considered as 100% deoxyribose oxidation without addition of AEVA. The percentage inhibition of the degradation was calculated according to the equation I = (A_0_-A_1_/A_0_) × 100, where A_0_ is the absorbance of the control and A_1_ is the absorbance of test sample. Trolox was used as positive control.

#### Quenching of superoxide radical

The SOD activity was measured following the method as described Manna at el. [23]. The reaction mixture contained sodium pyrophosphate buffer (0.052 mM, pH 8.3), NBT (300 μM,), PMT (186 μM,), and different concentrations (1.25, 2.5, 5, 7.5, 10, 12.5, and 15 μg/μL) of AEVA. The reaction was started by adding NADH (780 μM). The reaction mixture was incubated for 90 s at 30°C. After incubation, 1 mL of glacial acetic acid was added to stop the reaction and the absorbance was read at 560 nm. Results were expressed as percentage inhibition by using the equation, I = (A_0_-A_1_/A_0_) × 100, where A_0_ is the absorbance of the blank and A_1_ is the absorbance of test sample. Ascorbic acid was used as a positive control.

#### Assessment of the effect of AEVA on the activities of antioxidant enzymes using recombinant proteins and human plasma

For the assessment of the effect of AEVA on the activities of different antioxidant enzyme, various concentrations of AEVA (1.25, 2.5, 5, 7.5, and 10 μg/μL) were incubated with 100 μL of either rGST (36 μg/mL) or rCAT (100μg/mL) for 2 h at 37°C. For the assessment of the effect of AEVA on the activities of antioxidant enzymes in plasma, 100 μL of human plasma (collected from normal subject) was incubated with different concentrations of AEVA (1.25, 2.5, 5, 7.5, and 10μg/μL) for 2 h at 37°C. We have also examined the activities of the antioxidant enzymes in the extract itself. For this assay, 100 μL of phosphate buffer was incubated with different concentrations of AEVA (1.25, 2.5, 5, 7.5, and 10μg/μL) for 2 h at 37°C. After incubation, 10 μL samples from each group were used for the assessment of different antioxidant enzymes activities.

### Cell culture studies

#### Human Monocyte Cell Culture

The human THP-1 monocyte cell line was obtained from National Centre for Cell Sciences (Pune, India). These cells were maintained at 37°C in RPMI 1640 medium containing 5.5mM glucose, 10% (v/v) heat inactivated FBS, 100 units/mL penicillin, 100 μg/mL streptomycin,12 mM sodium carbonate, 25mM HEPES, and 2 mM L-glutamine in a humidified atmosphere containing 5% (v/v) CO_2_ [24]. For treatments, cells were washed once in plain RPMI before being suspended in fresh medium (complete) containing serum and other supplements.

#### Treatment of monocytes with H_2_O_2_, AEVA, and NAC

Cells were treated with different concentrations of AEVA (0.4, 0.8, 1.2 μg/μL) or NAC (positive control, 200 μM) for 2 h followed by H_2_O_2_ (25 μM) exposure for next 2 h. After treatment, cells were washed three times in PBS and lysed in radio immuno-precipitation assay (RIPA) buffer (50 mM Tris in pH 8, 150 mM NaCl, 1%NP‐40, 0.5% deoxycholic acid, and 0.1% SDS) supplemented with protease inhibitors (1mM PMSF, 5 mg/mL leupeptin, 2 mg/mL aprotinin, and 1mM EDTA). Lysates were cleared by centrifugation, total protein concentrations were determined using the BCA assay as per manufacturer’s protocol (Pierce/Thermo Scientific, Rockford, IL), and stored at -80°C for the assessment of antioxidant enzyme activities. Cell viability was determined using the Alamar Blue reduction bioassay (Himedia, India). This method is based upon Alamar Blue dye reduction by live cells.

### Antioxidant Enzyme Activity Assay

#### GST Activity Assay

GST catalyzes the conjugation reaction of the reduced form of glutathione in the first step of mercapturic acid synthesis. GST activity was measured by the method reported before [22]. The reaction mixture contained phosphate buffer (50 mM), CDNB (200 mM), GSH (200 mM), and 10 μL samples. The reaction was carried out at 37°C and the increase in absorbance of the conjugate of GSH and CDNB was monitored at 340 nm using microplate reader. A blank was run in absence of the sample. One unit of GST activity is defined as 1 μmol of product formation per minute per mL sample.

### CAT Activity Assay

The enzyme, CAT catalyzes the decomposition of H_2_O_2_ into H_2_O and O_2_. The CAT activity was measured by the method of Manna et al. [22]. The reaction mixture was consisted of 5 mM H_2_O_2_ and 10 μL sample. The breakdown of peroxide was continuously monitored at 240 nm for a specified period of time. One unit of CAT activity is defined as 1μmol conversion of H_2_O_2_ per minute per mL sample.

### Intracellular ROS Levels detection

Fluorescent dye DCFDA (2´,7´-dichlorofluoresceindiacetate) was used to measure the production of intracellular reactive oxygen species (ROS) levels [25]. The cells were treated AEVA (0.8 μg/μL) or NAC (200 μM) for 2 h followed by H_2_O_2_ (25 μM) exposure for the next 2 h. After treatment cells were washed with PBS and then incubated with 5μM DCFDA in PBS containing 4% FBS at 37°C for 30 min in the dark. After incubation cells were washed with PBS, centrifuged at 12,000 g for 10 min at 4°C, and mounted onto microscope slides using mounting medium. The images were taken by using the fluorescence microscope (Leica DM3000LED).

#### Statistical analysis

Data were analyzed statistically using ANOVA with Sigma Plot statistical software v13 (Jandel Scientific, San Rafael, CA). All groups were compared using the Student-Newman-Keuls *post hoc* analysis method. The *p* value less than 0.05 was considered to be statistically significant.

## Results

### Proximate compositions dietary principles in *Vespa affinis* L.

The moisture content and the nutritional value of *Vespa affinis* L. pupae were shown in Table 1.

**Table 1:** Proximate composition of nutritional value in *Vespa affinis* L. values were expressed per 100 g of pupae.

Nutrients	Pupae
Moisture (%)	79.59 ± 0.84
Protein (g)	25.20 ± 1.39
Lipids (g)	11.6 0± 0.92
Ash (g)	1.24 ± 0.07
Carbohydrate (g)	13.20± 1.28
Thiol (M)	211.42± 6.83

All values are mean ± SE (n = 5)

### Chemical profiling of AEVA

**Fig 1:**
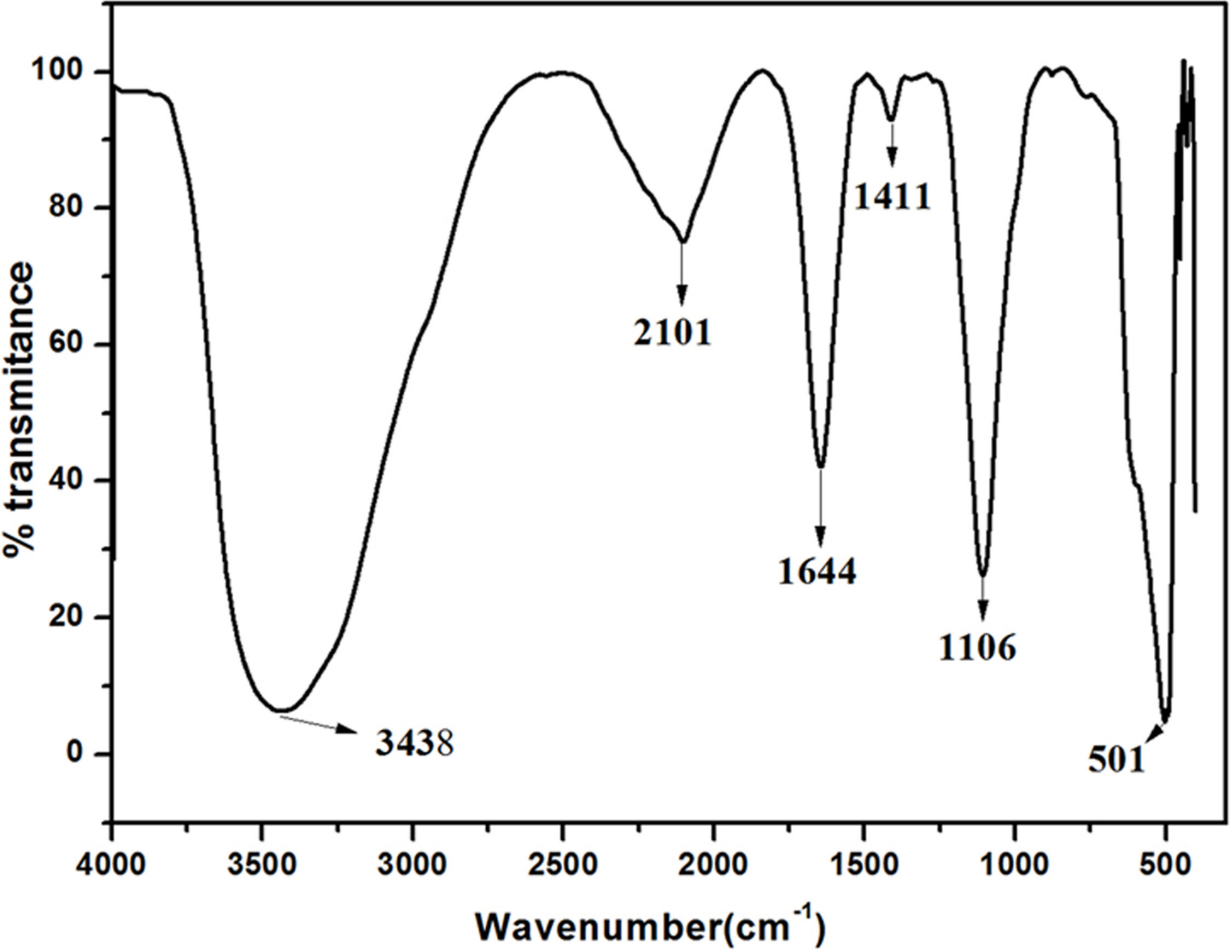
Infrared Spectrum analysis of AEVA.

**Fig 2:**
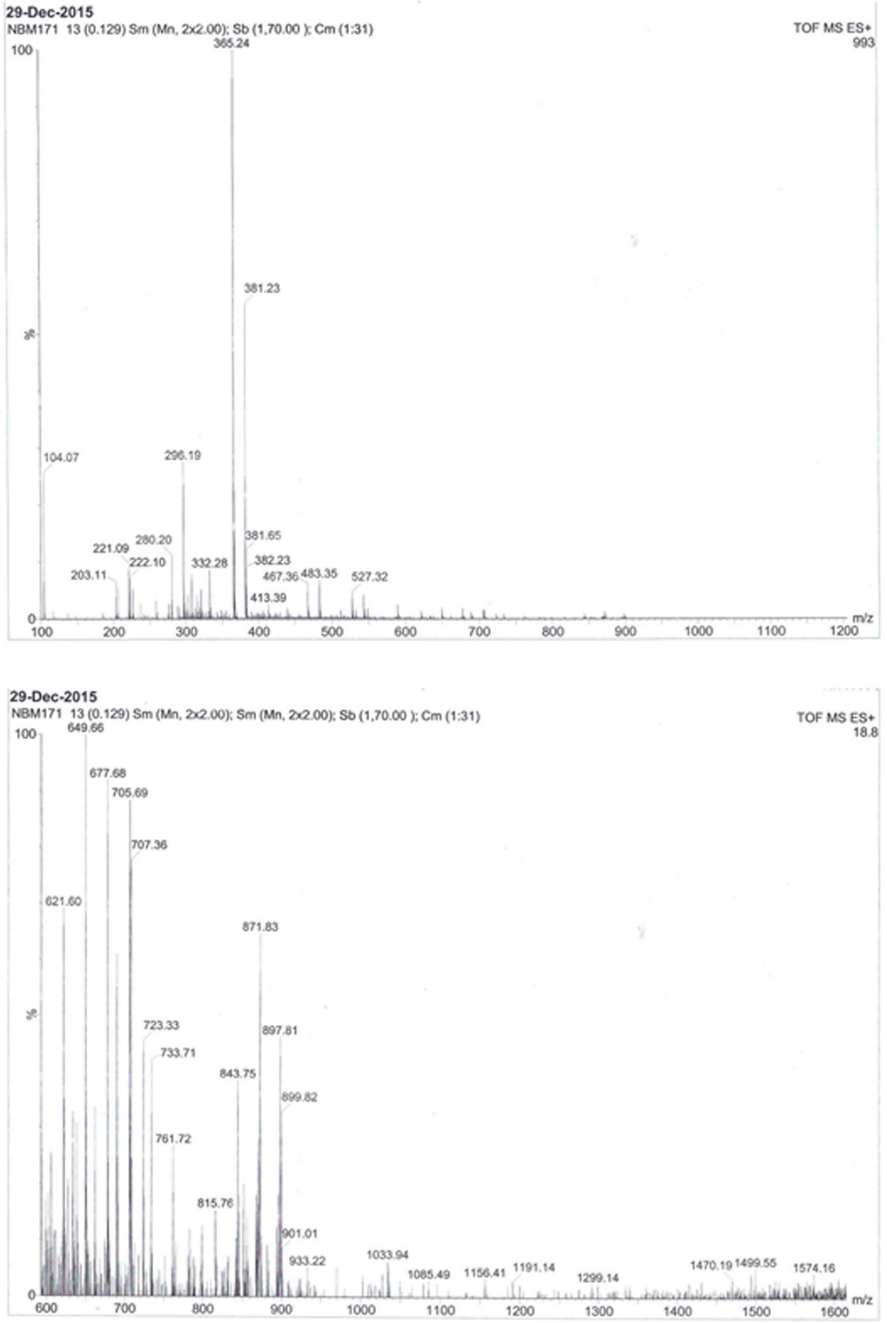
Mass Spectrum analysis of AEVA.

The IR spectrum analysis of AEVA demonstrates the presence of different functional groups ranging from O-H (3438 cm^-1^), C = C (1644 cm^-1^), C-O (1106 cm^-1^), and C-H (501 cm^-1^) (Fig 1). The MS analysis of AEVA represent the percentage of different components with m/z values ranging from 100–600 (upper panel) and 600–1600 (lower panel) (Fig 2). Both the IR and MS spectrum represent the chemical fingerprint of AEVA.

### Free radical scavenging activities of AEVA in cell free system

The free radical scavenging activities of AEVA were investigated using DPPH, hydroxyl, and superoxide radicals in cell free system.

### DPPH radical scavenging activity

**Fig 3:**
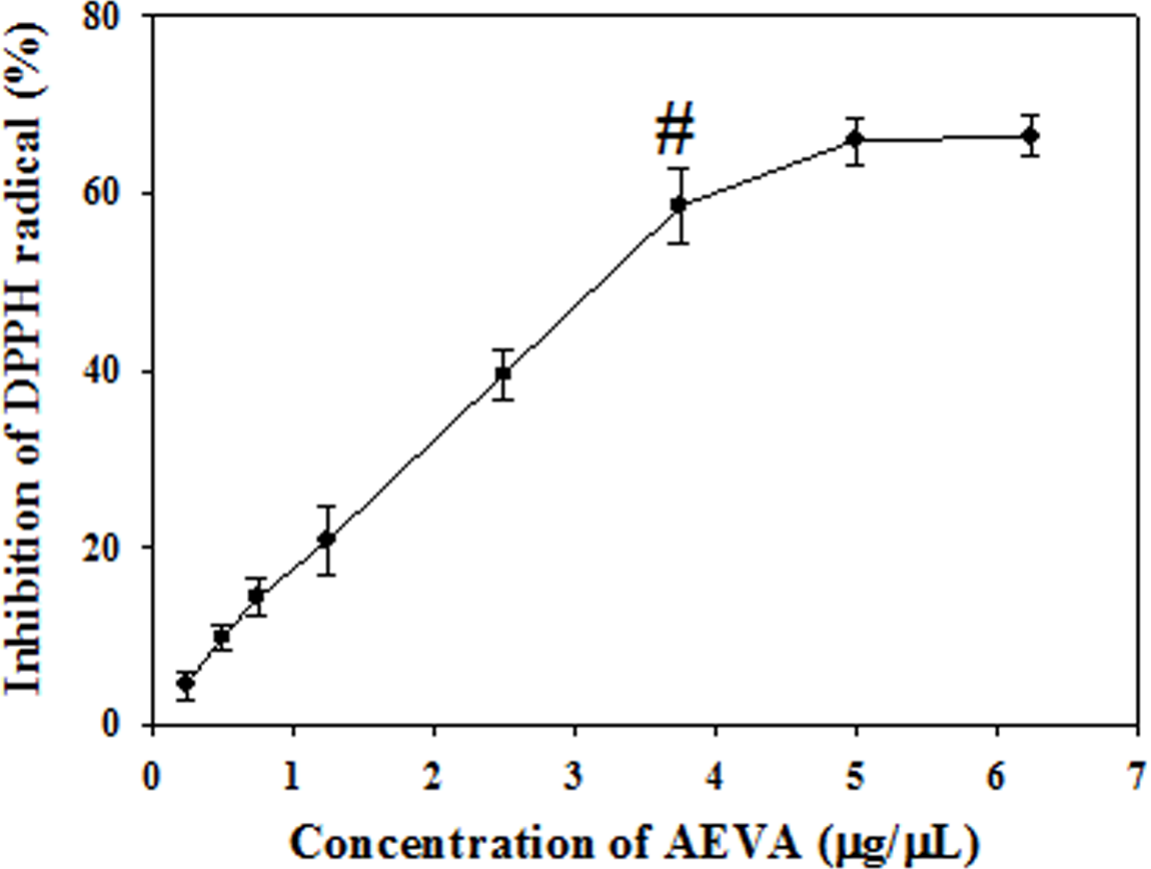
DPPH radical scavenging activity of AEVA. Graph is obtained by plotting different concentrations of AEVA (0.25, 0.5, 0.75, 1.25, 2.5, 3.75, 5, and 6.25 μg/μL) against the percent inhibition. “#” indicates the optimum concentration of AEVA at which it exhibits maximum inhibition. Values are mean ± SE (n = 4).

Fig 3 demonstrates the DPPH radical scavenging activity of AEVA. Results suggest that with the increasing concentration of AEVA the inhibition of DPPH radical increases and the maximum inhibition was observed at a concentration of 3.75 μg/μL AEVA. Thus it may be speculated that active principles of AEVA retain free radical scavenging activity.

### Hydroxyl radical scavenging activity

**Fig 4:**
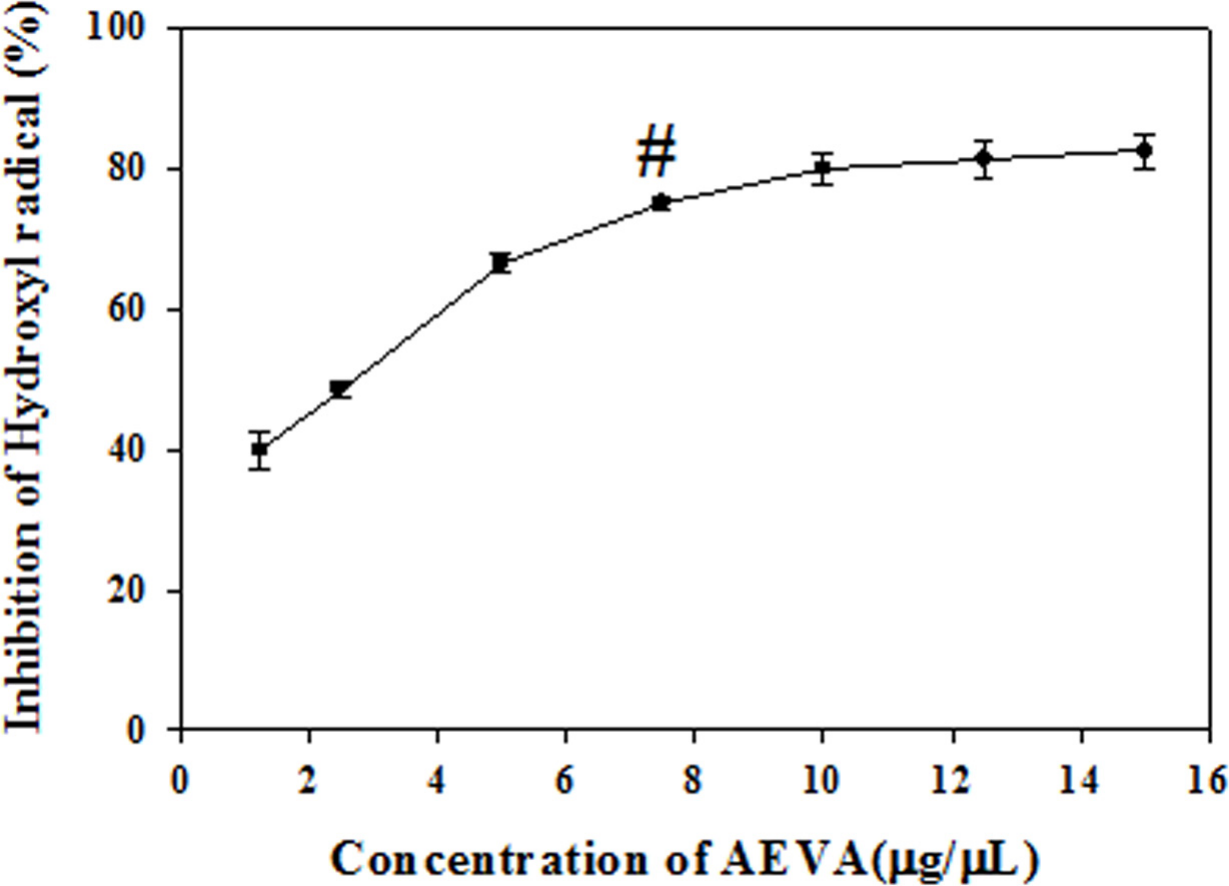
Hydroxyl radical scavenging activity of AEVA. Curve is obtained by plotting various concentrations of AEVA (1.25, 2.5, 5, 7.5, 10, 12.5, and 15 μg/μL) against percent inhibition. “#” indicates the optimum concentration of AEVA at which it exhibits maximum inhibition. Values are mean ± SE (n = 4).

The hydroxyl radical scavenging activity of AEVA has been examined in cell free system using Fe^3+^/Ascorbate/EDTA/H_2_O_2_ reagents and the results have been demonstrated in Fig 4. It has been observed that AEVA can inhibit the formation of hydroxyl radical in a dose-dependent manner and the maximum inhibition was observed at a concentration of 7.5μg/μL AEVA.

### Superoxide radical scavenging activity

**Fig 5:**
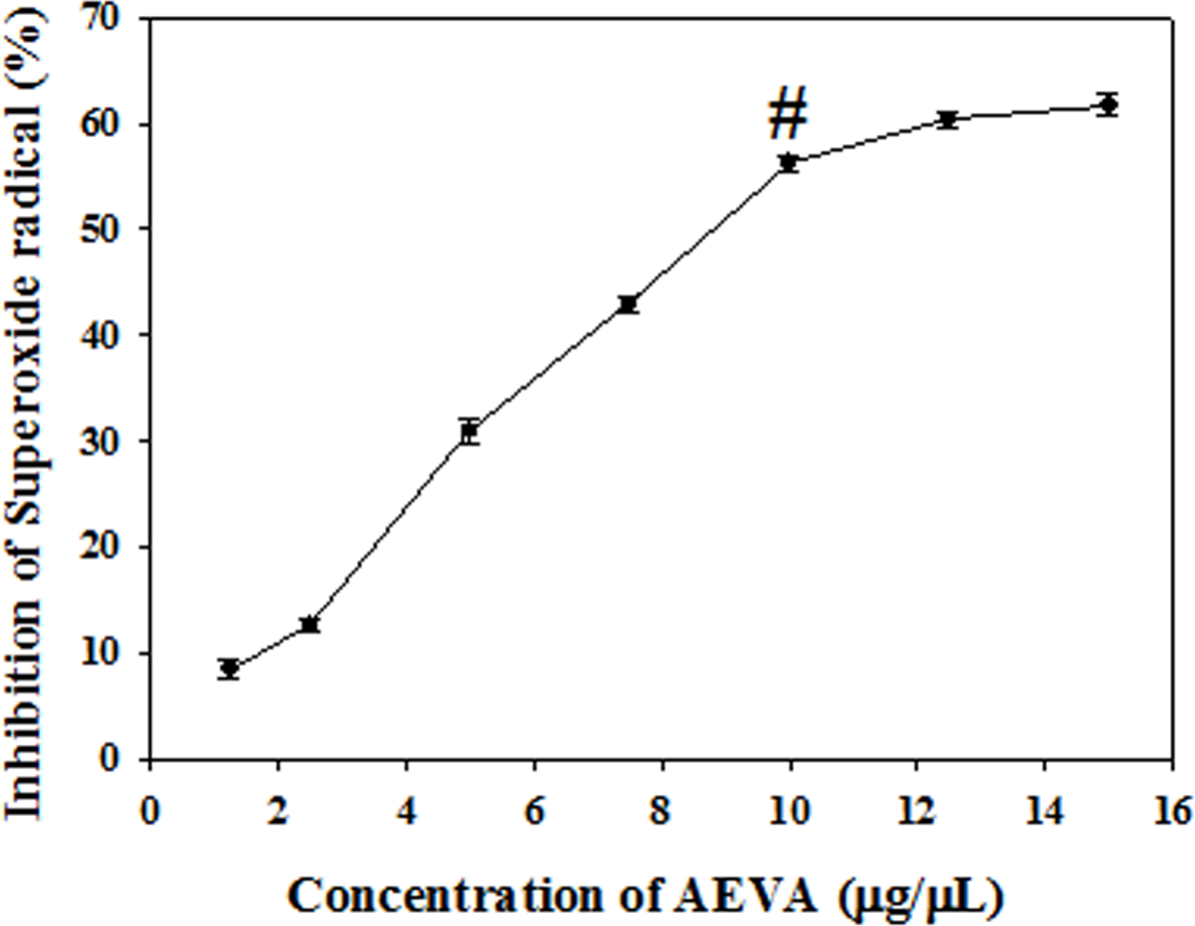
Superoxide radical scavenging activity of AEVA. Graph is obtained by plotting different concentrations of AEVA (1.25, 2.5, 5, 7.5, 10, 12.5, and15μg/μL) with respect to percent inhibition. “#” indicates the optimum concentration of AEVA at which it exhibits maximum inhibition. Values are mean ± SE (n = 4).

In addition to DPPH and hydroxyl radical scavenging activities, AEVA has also shown the super oxide radical scavenging activity and the results are demonstrated in Fig 5. It has been observed that at dose of10 μg/μL, AEVA shows maximum inhibitory activity against the formation of superoxide radicals.

### Activities of antioxidant enzymes in AEVA

**Fig 6:**
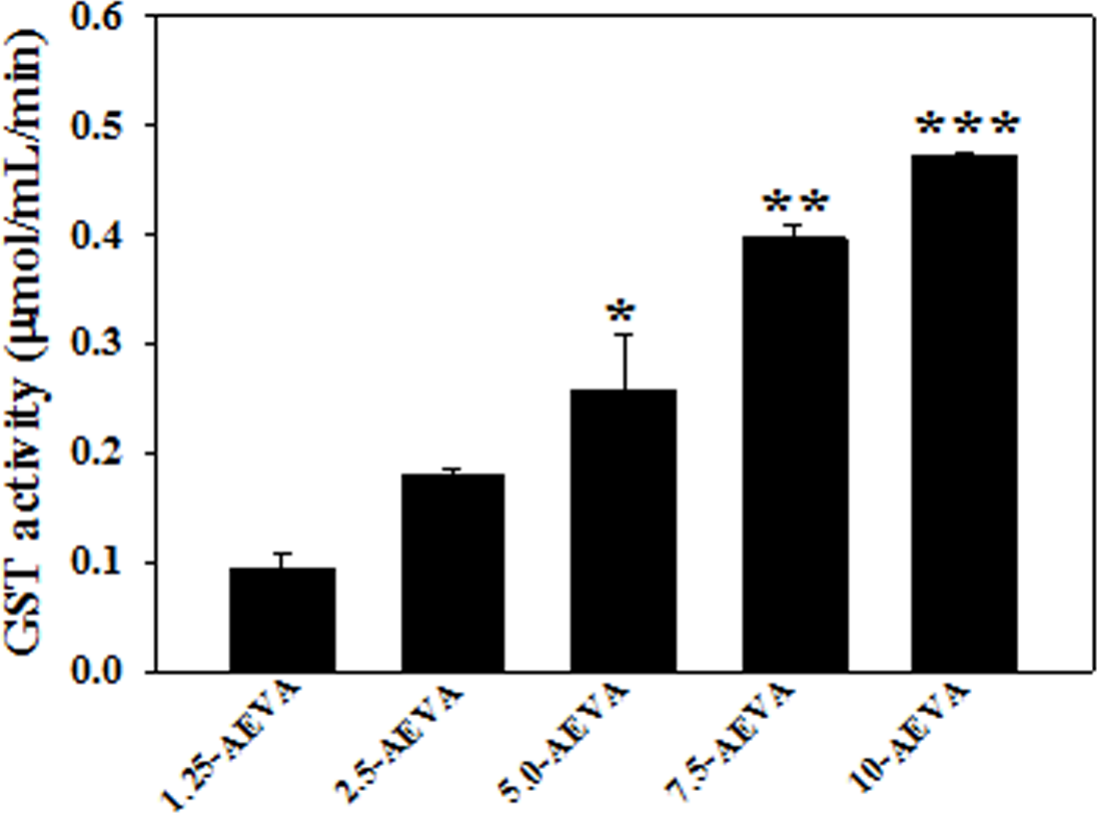
Dose dependent GST activity of AEVA. Graph is obtained by plotting different concentrations of AEVA (1.25, 2.5, 5, 7.5, and 10 μg/μL) with respect to enzyme activity. “*”, “**”, and “***” indicate the significant differences between the effect of 5, 7.5, and 10 μg/μL AEVA and its lower concentrations respectively (*p*< 0.05). Values are mean ± SE (n = 4).

**Fig 7:**
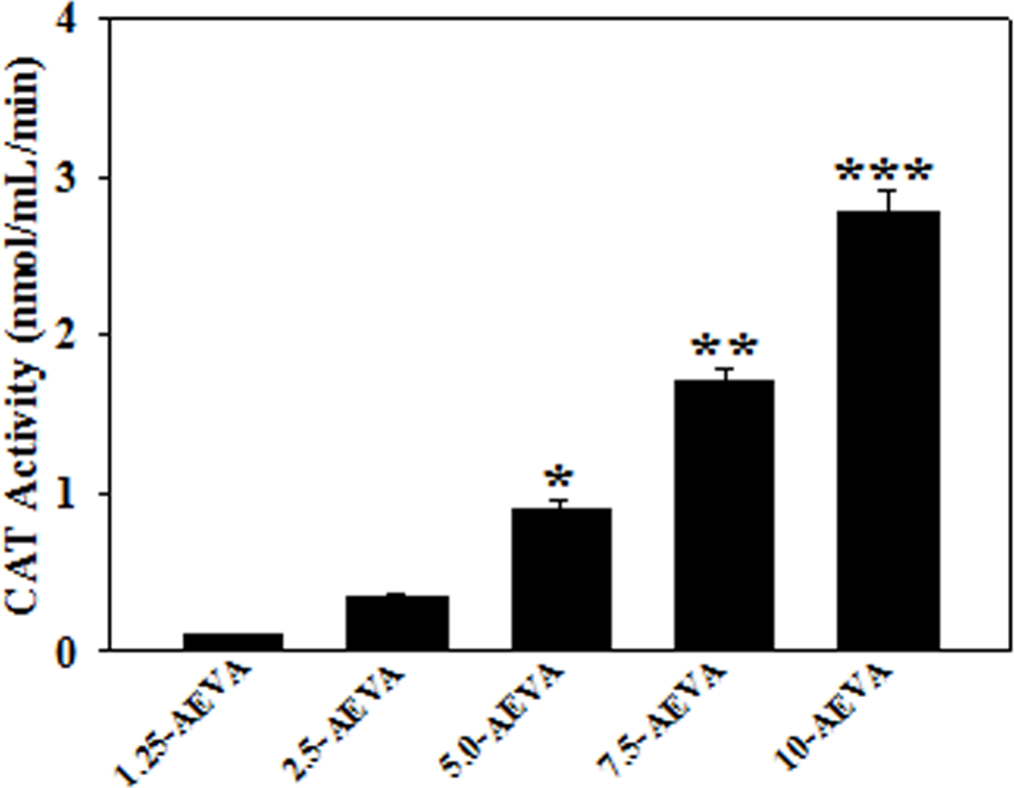
Dose dependent CAT activity of AEVA. Graph is obtained by plotting different concentrations of AEVA (1.25, 2.5, 5, 7.5, and 10 μg/μL) with respect to enzyme activity. “*”, “**”, and “***” indicate the significant differences between the effect of 5, 7.5, and 10 μg μL^-1^ AEVA and its lower concentrations respectively (*p*< 0.05). Values are mean ± SE (n = 4).

The activities of the antioxidant enzymes, namely GST and CAT have been examined in the AEVA. Figs 6 and 7 demonstrate that at a dose of 10 μg/μL, AEVA shows a significant GST and CAT enzyme activities compared to the lower concentrations (1.25, 2.5, and 5 μg/μL).

### Effect of AEVA on antioxidant enzyme activities using recombinant proteins

**Fig 8:**
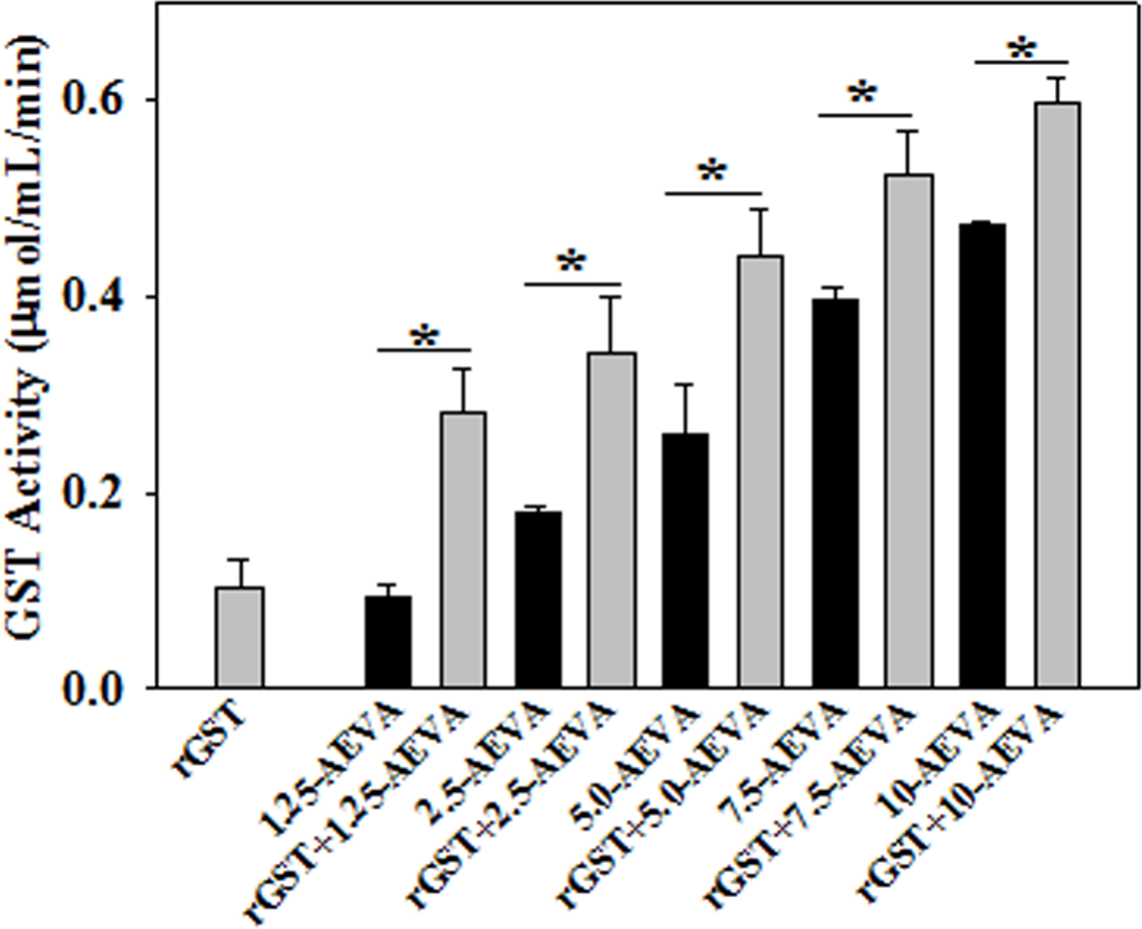
Dose dependent effect of AEVA on GST activity using recombinant GST (rGST). 100 μL of rGST (36 μg/mL) was incubated with different concentration of AEVA (1.25, 2.50, 5.00, 7.50, and 10.00 μg μL^-1^) for 2 h at 37°C. “*” indicates the significant difference between “AEVA alone” and “rGST+ AEVA” at the respective concentration of AEVA (p*< 0.05). Values are mean ± SE (n = 4).

**Fig 9:**
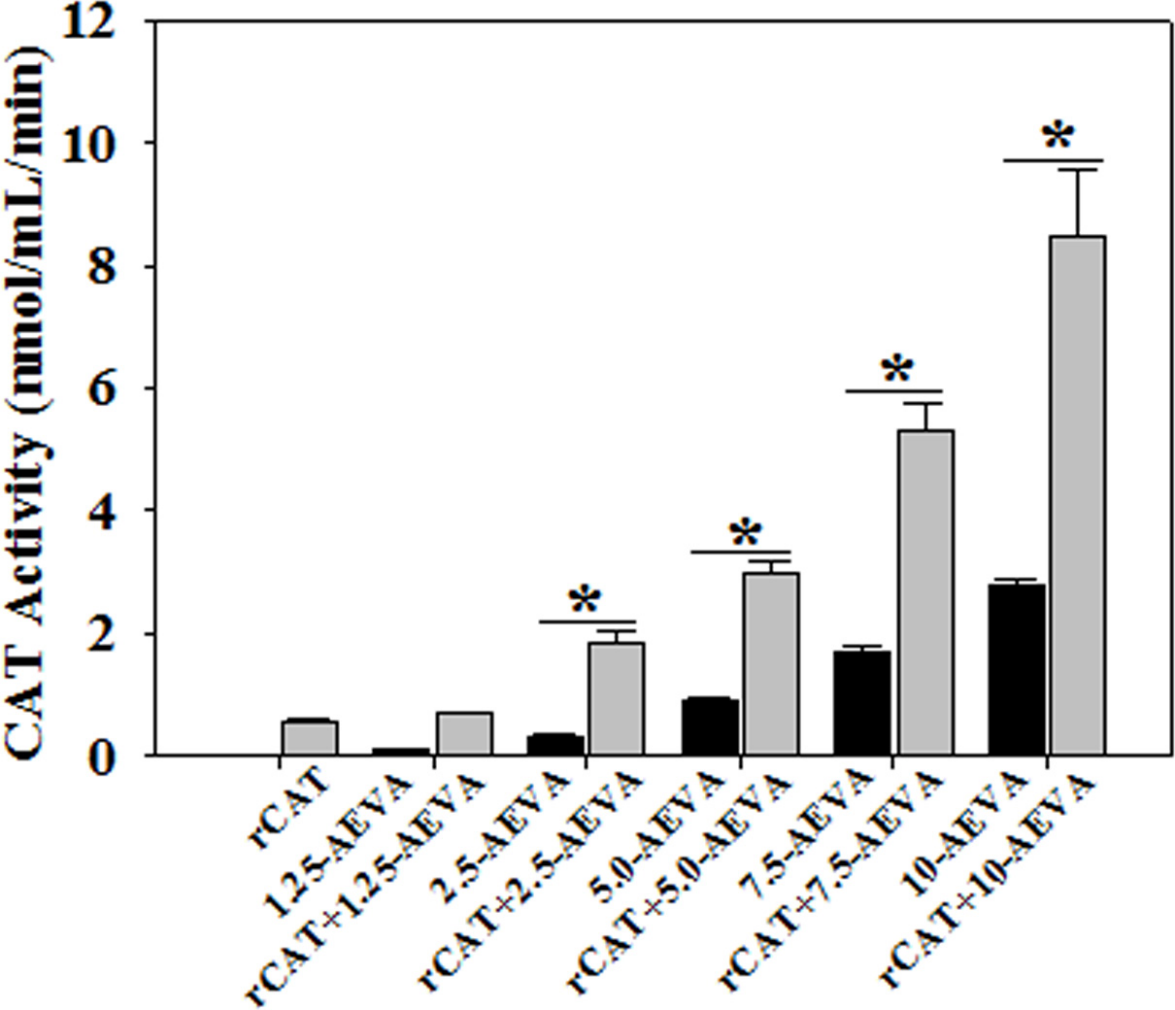
Dose dependent effect of AEVA on CAT activity using recombinant CAT (rCAT). 100 μL of rCAT (100 μg/mL) was incubated with different concentration of AEVA (1.25, 2.50, 5.00, 7.50, and 10.00 μg μL^-1^) for 2 h at 37°C. “*”indicates the significant difference between “AEVA alone” and “rCAT + AEVA” at the respective concentration of AEVA (p*< 0.05). Values are mean ± SE (n = 4).

The effect of AEVA on the activities of the antioxidant enzymes, such as GST and CAT has been investigated using recombinant proteins. Figs 8 and 9 demonstrate the effect AEVA on the enzyme activities of GST and CAT using rGST and rCAT, respectively. It has been observed that incubation of AEVA at a dose of 1.25μg/μL and 2.5μg/μL onwards significantly increased the activities of recombinant enzymes, rGST and rCAT respectively compared to those seen in corresponding enzyme alone or AEVA itself. This suggests a prophylactic role of AEVA in stimulating the activities of antioxidant enzyme.

### Effect of AEVA on GST and CAT enzyme activities in human plasma

**Fig 10:**
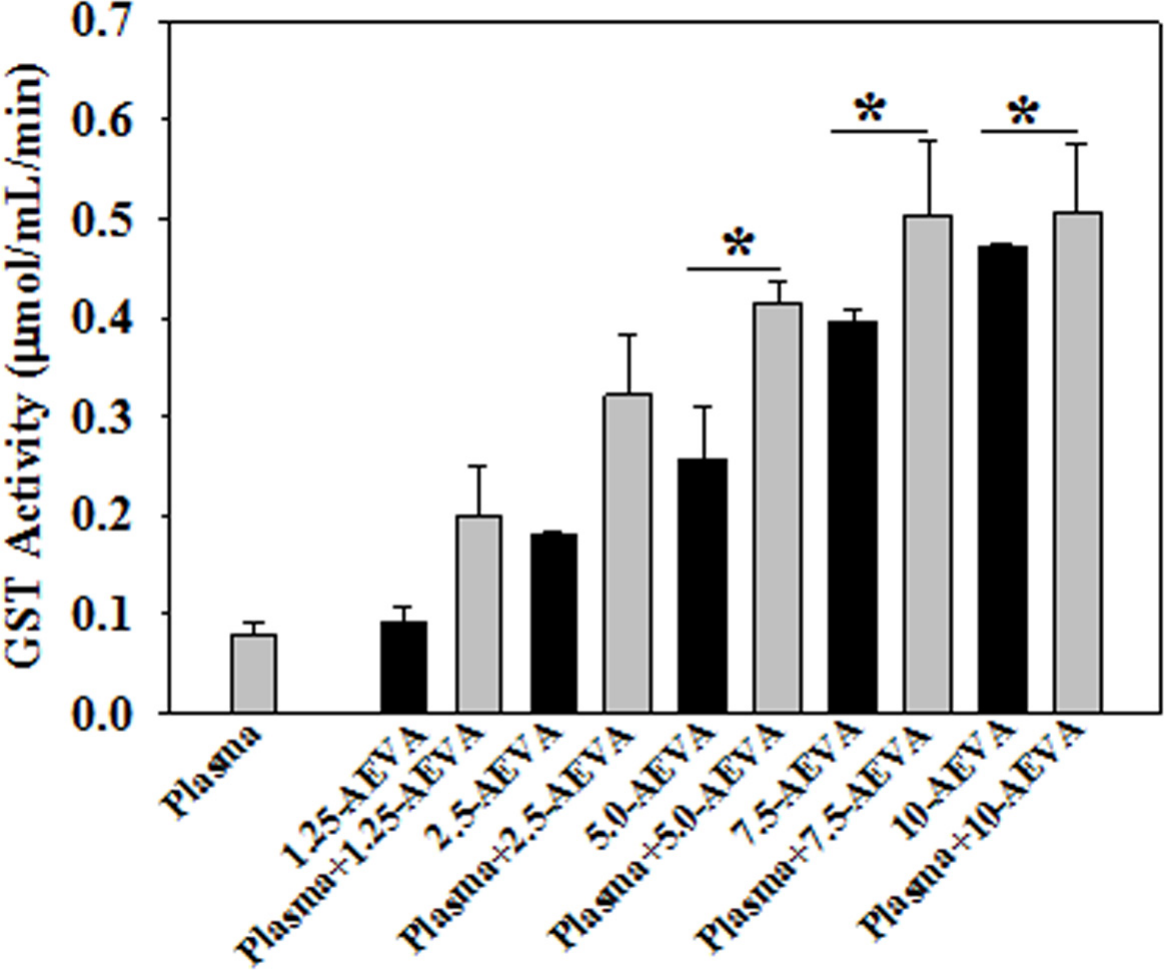
Effect of AEVA on GST activity in human plasma. 100 μL of human plasma (collected from normal subject) was incubated with different concentration of AEVA (1.25, 2.50, 5.00, 7.50, and 10.00 μg μL^-1^) for 2 h at 37°C. “*” indicates the significant difference between “AEVA alone” and “plasma + AEVA” at the respective concentration of AEVA (*p*< 0.05). Values are mean ± SE (n = 4).

**Fig 11:**
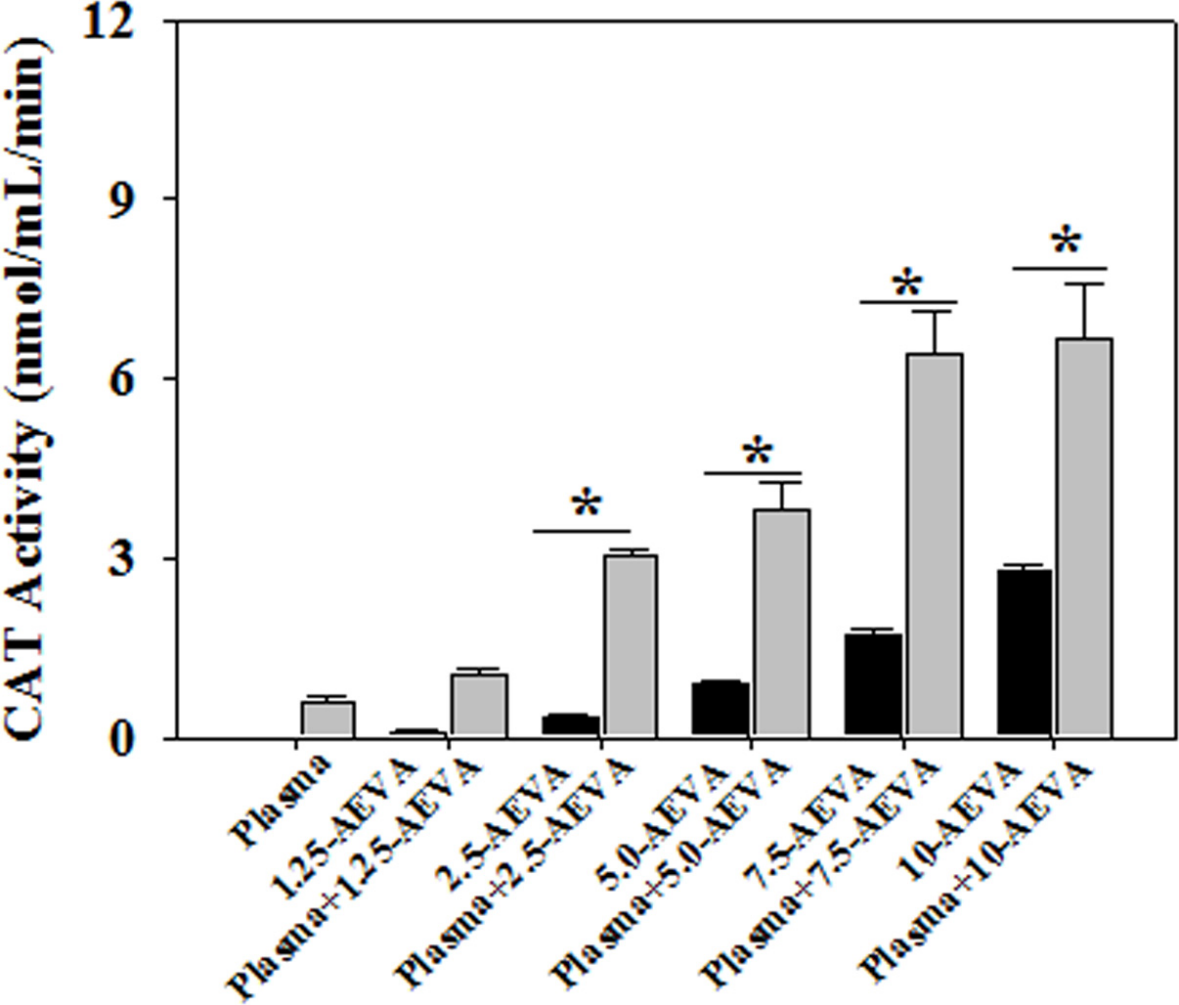
Effect of AEVA on CAT activity in human plasma. 100 μL of human plasma (collected from normal subject) was incubated with different concentration of AEVA (1.25, 2.50, 5.00, 7.50, and 10.00 μg μL^-1^) for 2 h at 37°C. “*” indicates the significant difference between “AEVA alone” and “plasma + AEVA” at the respective concentration of AEVA (*p*< 0.05). Values are mean ± SE (n = 4).

The beneficial role of AEVA in stimulating the activities of antioxidant enzymes has also been investigated using human plasma collected from healthy individuals. Figs 10 and 11 demonstrate the effect AEVA on GST and CAT enzyme activities in human plasma. Results suggest that incubation of AEVA at a dose of 5 μg/μL and 2.5 μg/μL onwards significantly increased the enzyme activities of GST and CAT respectively compared to those seen in plasma or AEVA alone. In addition to the studies with recombinant proteins, these results also suggest the beneficial role of AEVA in stimulating the activities of antioxidant enzymes.

### Effect of AEVA on GST and CAT activity in monocytes

**Fig 12:**
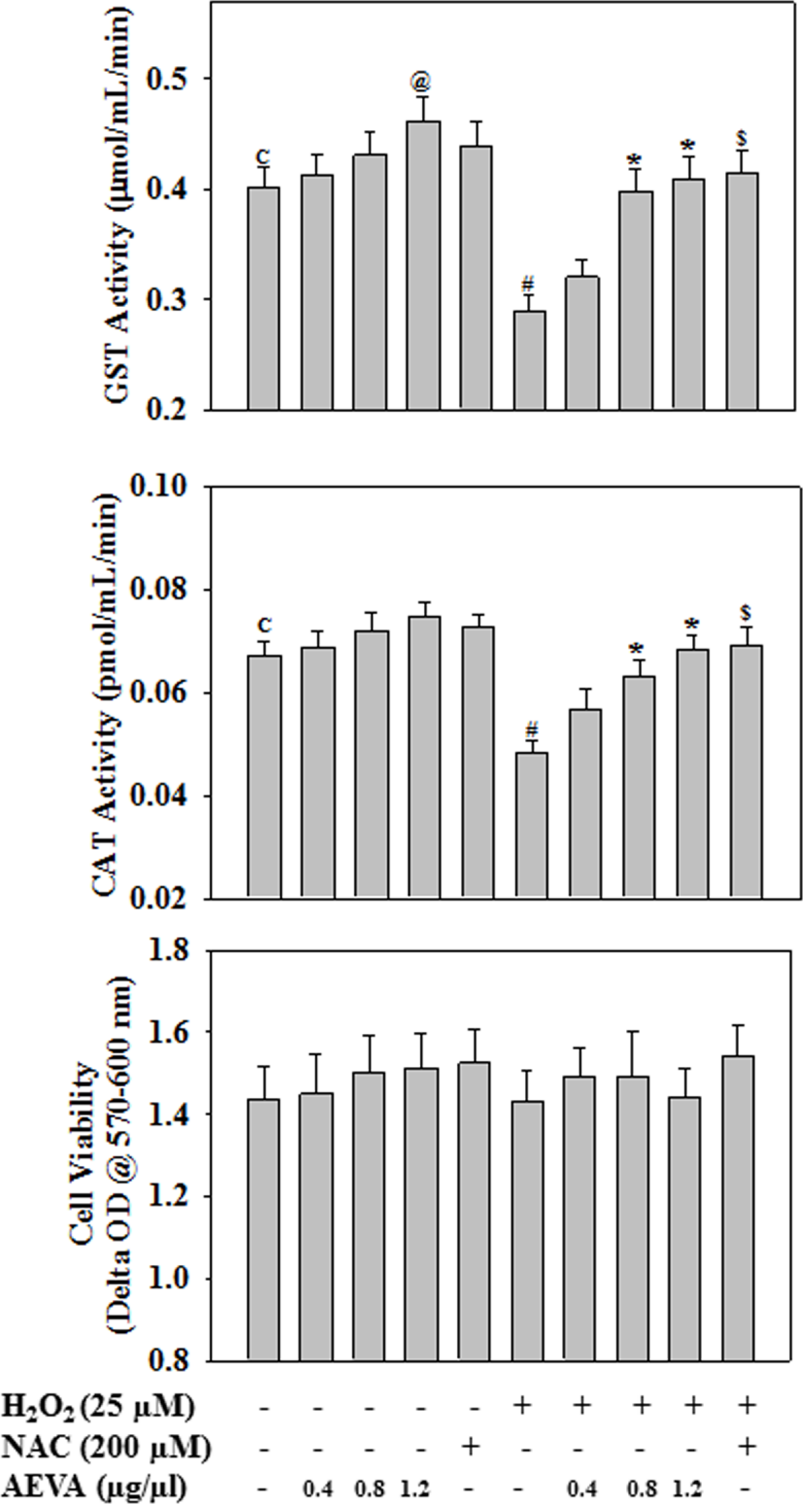
Effect of AEVA or NAC (N-acetyl cysteine, positive control) on GST and CAT enzyme activity in THP-1 monocyte cells. Cells were treated with different concentrations of AEVA (0.4, 0.8, and 1.2 μg/μL) or NAC (200 uM) for 2 h followed by H_2_O_2_ exposure (25 μM) for next 2 h. “*” indicates the significant difference between “H_2_O_2_” and “AEVA+ H_2_O_2_”-treated group(*p*< 0.05); “$” indicates the significant difference between “H_2_O_2_” and “NAC+ H_2_O_2_”-treated group; “@” indicates the significant difference between “control (C)” and “AEVA (1.2 μg/μL)”-treated group. Values are mean ± SE (n = 4).

The antioxidant potential of AEVA has also been investigated using human THP-1 monocyte cells exposed to a well-known pro-oxidant H_2_O_2_. NAC, a standard antioxidant was used as a positive control. In case of radical scavenging assay in cell free system, AEVA was showing significant radical scavenging activity at a dose of 1.25 μg/μL. In order to investigate the antioxidant role of AEVA in cellular system, monocytes were treated with AEVA at a dose of 1.25 μg/μL and two lower concentrations (0.4 and 0.8 μg/μL). Results demonstrate that H_2_O_2_ exposure caused a significant decrease in GST and CAT activities in monocytes. However, treatment with AEVA dose dependently (0.4, 0.8, and 1.2 μg/μL) increased the GST and CAT activities against the H_2_O_2_ exposure (Fig 12). Supplementation with NAC (200 μM) also increased the GST and CAT activity against the H_2_O_2_ exposure and the effect is comparable with what observed in AEVA treatment at a dose of 0.8 and 1.2 μg/μL. Treatment with AEVA also increased the GST activity significantly at a dose of 1.2 μg/μL compared to control cells. Different treatment did not cause any change in cell viability. The outcome of the cell culture study also validates the antioxidant potential of AEVA.

### Effect of AEVA on intercellular ROS production

**Fig 13:**
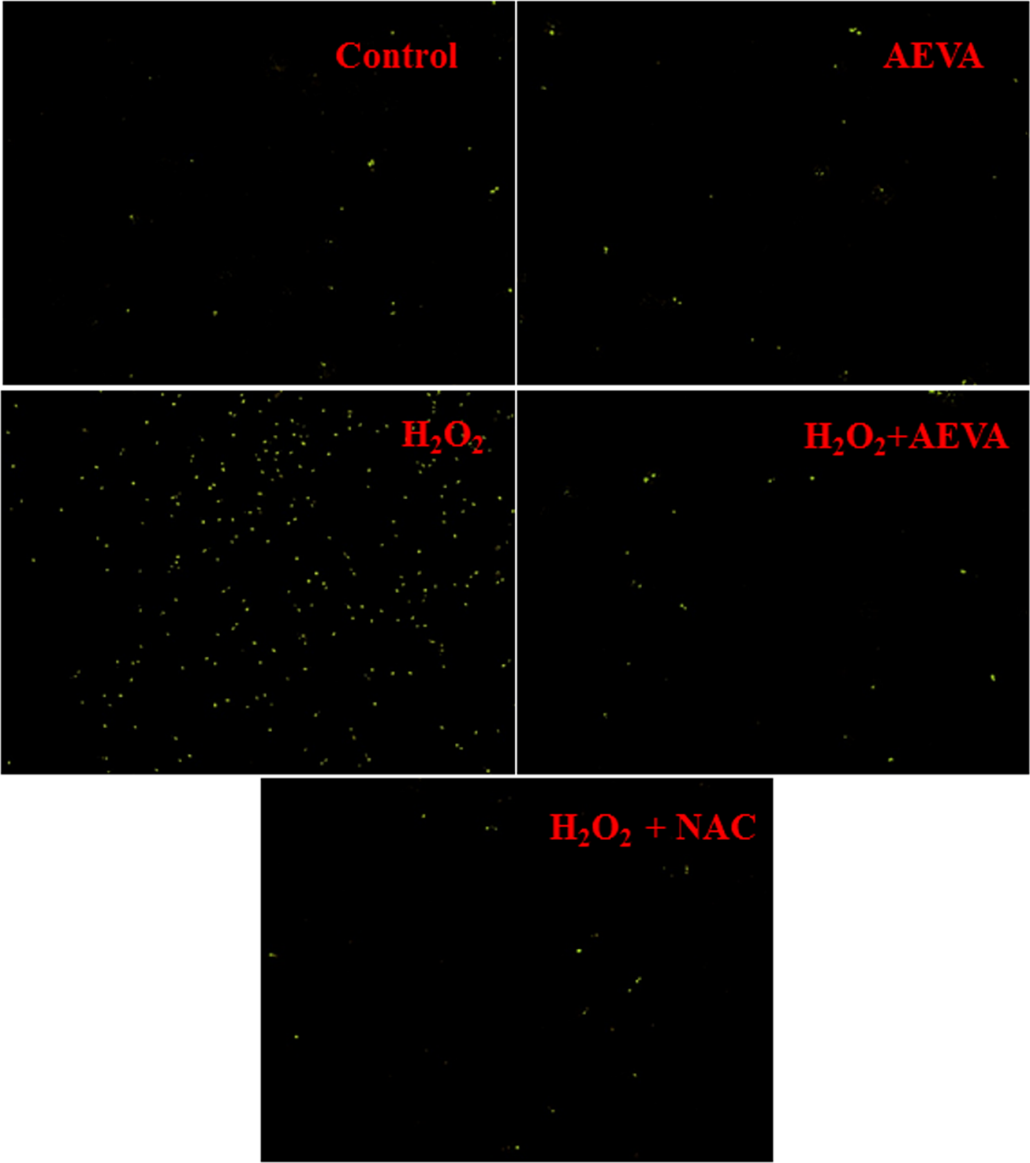
Effect of AEVA or NAC (N-acetyl cysteine) on the levels of intracellular reactive oxygen species (ROS) in H_2_O_2_-treated THP-1 monocyte cells. Cells were treated with AEVA (0.8 μg/μL) or NAC (200 μM) for 2 h followed by H_2_O_2_ (25 μM) for next 2 h. The intercellular ROS production was measured using 5μM DCFDA in PBS containing 4% FBS at 37°C for 30 min in the dark.

Elevated ROS production plays an important role in the development of oxidative stress. The present study examined the effect of AEVA on the intracellular ROS production in THP-1 monocyte cells treated with H_2_O_2_ (25 μM) (Fig 13). The result suggests that the treatment with AEVA at a dose of 0.8 μg/μL significantly decrease the intercellular ROS production and the results are also comparable to what observed in positive control, NAC-treated cells.

## Discussion

People around the world are traditionally using insects for the treatment of different types of diseases [5, 26, 27]. *Vespa sp*. is commonly taken edible insect by various tribes and communities in North-East Indian [6, 12, 13]. A very limited study reported the traditional uses of this edible insect for the treatment of arthritis complication [5]. However, any mechanistic study has not been carried out to examine the beneficial property of this insect. The present study was designed to appraise the molecular mechanism underlying the medicinal benefit of the aqueous extract of *Vespa affinis* L. (AEVA).

Elevated oxidative stress plays an important role in the pathogenesis of several health disorders, such as diabetes, cardiovascular dysfunction, arthritis, etc. [28, 29]. Altindag *et al*. reported total antioxidant and total oxidative status in plasma levels are higher in patients with arthritis complications compared to those seen in healthy subjects [29]. Various studies suggest the role of oxidative in the pathogenesis of diabetes and its complications [28]. Oxidative stress occurs when the generation of reactive oxygen species (ROS, such as superoxide radical, hydroxyl radical, hydrogen peroxide, etc.) exceeds the system’s ability to neutralize and eliminate them. Various enzymatic and non-enzymatic molecules have been developed by the cell to cope up with the ROS and other free radicals. However, a decrease in the intracellular antioxidant defense caused by the disturbance in the production, distribution, or by an over-abundance of ROS from an environmental or behavioral stressor leads to the oxidative stress pathophysiology. If not regulated properly, the excess ROS can initiate lipid peroxidation in cell membrane, cause the destruction of cellular components, inhibit the normal cellular function, and finally lead to cell death [30].

In order to investigate the radical scavenging activities of AEVA, we initially examined the DPPH radical scavenging activity of AEVA. DPPH is a well-known stable free radical commonly used to screen the radical scavenging activity of a substance in cell free system [31]. Our results demonstrate a dose-dependent increase in the inhibition of DPPH radical by AEVA and the maximum inhibition was observed at a concentration of 3.75 μg/μL AEVA. In addition to the DPPH radical, this study also investigated both the hydroxyl and super oxide radical scavenging potential of AEVA in cell free system using Fe^3+^/Ascorbate/EDTA/H_2_O_2_ and PMT/NADH/NBT reagent systems, respectively. Results suggest that AEVA can inhibit the formation of both hydroxyl and superoxide radicals in a dose-dependent manner and the maximum inhibition was observed at a concentration of 7.5 μg/μL AEVA and 10 μg/μL AEVA, respectively.

The intracellular antioxidant defense system is comprised of different antioxidant enzymes, like SOD, CAT, GST, etc. together with the non-enzymatic substances, such as vitamin C, vitamin E, etc. and compounds containing thiol groups, like GSH, which are capable of reducing ROS or preventing their formation [30, 32]. CAT is one of the major antioxidant enzyme, which is involved in the detoxification of H_2_O_2_ via catalyzing the conversion of H_2_O_2_ into water and oxygen, while GST catalyzes a variety of reactions that detoxify endogenous as well as exogenous toxic compounds, including peroxidized lipids [33, 34].

**Fig 14:**
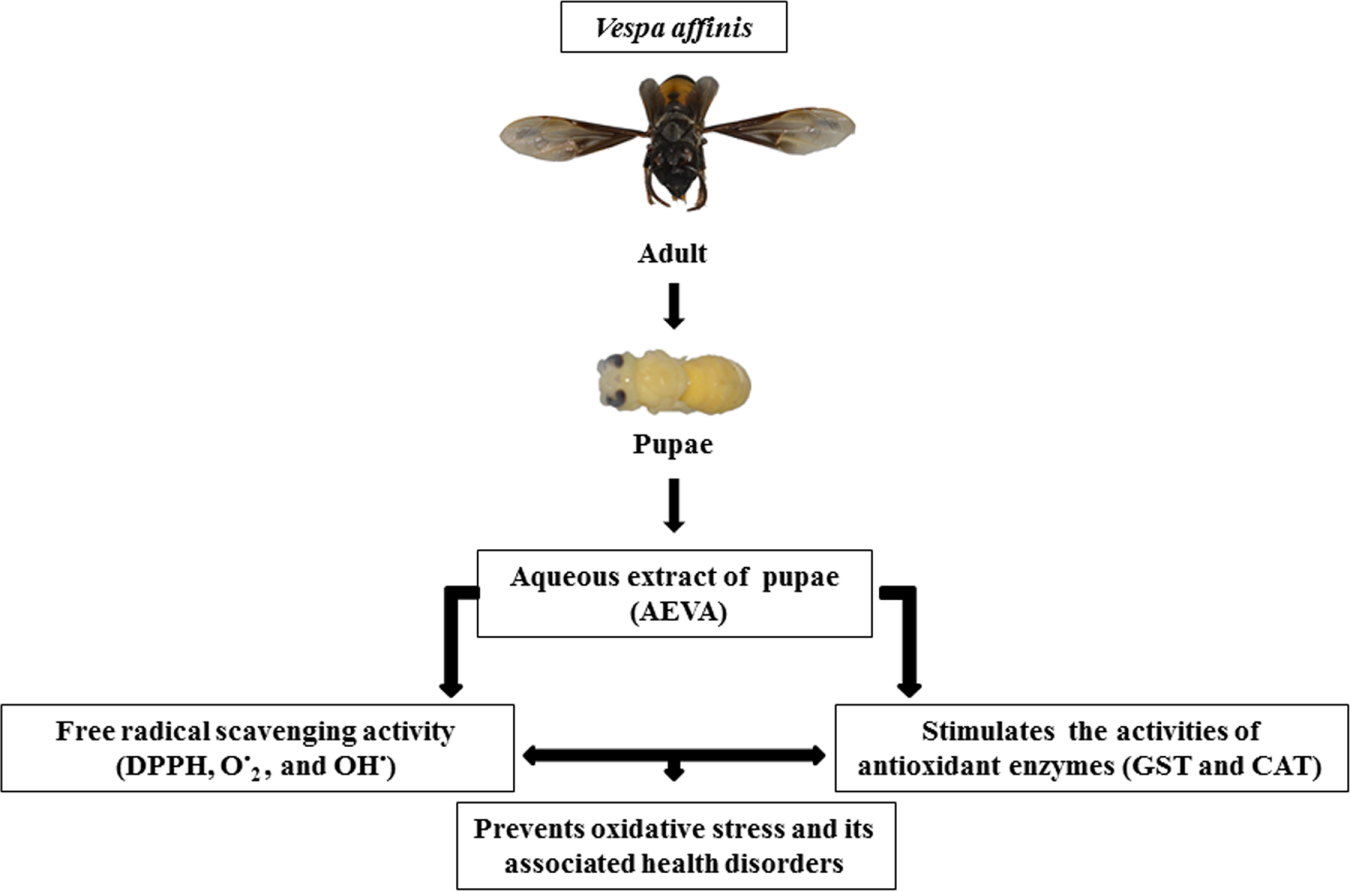
Flow diagram showing antioxidant potential of *Vespa affinis* L.

The present study demonstrates the presence of significant antioxidant enzymes activities, namely GST and CAT in AEVA, which may explain the inhibitory role of AVEA against the production of free radicals in cell free system. Further studies show that incubation of AEVA with the recombinant antioxidant enzymes, rGST and rCAT significantly increases the enzyme activities compared to those seen in recombinant enzyme alone or AEVA itself. Using human plasma we have also investigated the beneficial role of AEVA in stimulating the activities of antioxidant enzymes. Results suggest that incubation of AEVA with human plasma also increased the activities of GST and CAT compared to those seen in either plasma alone or AEVA itself. Using THP-1 monocyte cell culture model our study also demonstrates that treatment with a pro-oxidant, H_2_O_2_ caused a significant decrease in GST and CAT activity. However, supplementation with AEVA significantly increased the GST and CAT activity at a dose of 0.8 and 1.2 μg/μL in the H_2_O_2_-treated cells We have also investigated the intracellular radical scavenging activity of AEVA using DCFDA. Results suggest that AEVA can also prevent the H_2_O_2_-induced intracellular ROS production in monocytes. The antioxidant potential of AEVA is comparable to what observed in NAC, a standard antioxidant against H_2_O_2_ exposure. In our study we have also observed a significant amount of total thiol content in AEVA which may also explain its beneficial function against oxidative stress. Combining all, the effect of AEVA on the inhibition of free radical production and stimulation of the activities of antioxidant enzymes suggest a beneficial role of the extract against oxidative stress associated heath complications (Fig 14).

## Conclusion

This study for the first time demonstrates the antioxidant potential of the aqueous extract of an edible insect species commonly used in North-East India, which may mediate the therapeutic activities of the extract in oxidative stress-associated health disorders. Our future *in vivo* studies including animal model will validate the present observations. In addition, to have a clear picture, further investigations are required to identify and fully characterize the active principle(s) present in the extract and this is currently in progress.
